# Discrimination of Complex Human Behavior by Pigeons (*Columba livia*) and Humans

**DOI:** 10.1371/journal.pone.0112342

**Published:** 2014-11-07

**Authors:** Muhammad A. J. Qadri, Justin M. Sayde, Robert G. Cook

**Affiliations:** Department of Psychology, Tufts University, Medford, Massachusetts, United States of America; Bowling Green State Universtiy, United States of America

## Abstract

The cognitive and neural mechanisms for recognizing and categorizing behavior are not well understood in non-human animals. In the current experiments, pigeons and humans learned to categorize two non-repeating, complex human behaviors (“martial arts” vs. “Indian dance”). Using multiple video exemplars of a digital human model, pigeons discriminated these behaviors in a go/no-go task and humans in a choice task. Experiment 1 found that pigeons already experienced with discriminating the locomotive actions of digital animals acquired the discrimination more rapidly when action information was available than when only pose information was available. Experiments 2 and 3 found this same *dynamic superiority effect* with naïve pigeons and human participants. Both species used the same combination of immediately available static pose information and more slowly perceived dynamic action cues to discriminate the behavioral categories. Theories based on generalized visual mechanisms, as opposed to embodied, species-specific action networks, offer a parsimonious account of how these different animals recognize behavior across and within species.

## Introduction

Interpreting and categorizing the behavior of others is an essential social skill for humans. We have highly developed capacities for this function [1], [2]. With the discovery of mirror neurons in monkeys [3], a number of motor-based theories of human action recognition, embodied cognition, language, intentionality, and social cognition have been proposed [4]–[8]. One theory has pivoted around the notion that humans have an action observation network which is critically tied to the embodied simulation of the bodily movements of others and is key to understanding conspecific actions and intentions (e.g., [4]). Some suggest that this system uses evolved, species-specific motor-based knowledge to recognize actions by internally simulating or emulating observed actions [7], [9].

In contrast to these motor-based theories of action recognition are theories of action recognition based solely on visual mechanisms. Decades of computational vision research aimed at decoding the actions depicted in videos have identified a wide range of methods for this task (for a thorough overview, see [10]). Computational methods involving the extraction of local features, two-dimensional (2D) models, or various three-dimensional (3D) models have all succeeded at various levels, without using embodied motor representations or systems to recognize behavior. Biologically plausible models of vision-only action recognition have successfully modeled human and non-human primate performance in behavior recognition tasks [11], [12]. In a vision-only behavior recognition framework, the motor activations thought to confirm the existence of a mirror neuron system are the result of visuo-motor associations created during learning. Hence, the “mirror system” is a result of within-lifetime learning, not an evolutionary visuo-motor linkage necessary for action understanding [13].

The comparable theoretical analysis of action recognition in non-primates has proceeded more slowly due to the difficulty of controlling and experimentally manipulating animal behavior in natural situations when using recorded videos as stimuli [14], [15]. Concerns have also been raised regarding the naturalness or validity of the video presentations, with focuses on lower-level issues like color perception, flicker fusion, and motion perception and on higher-level concerns such as correspondence [16], [17]. These concerns generally center around whether the parameters for the displays are properly attuned for birds to see the display as we see it, like persistent shapes in smooth motion, and not as flickering shapes that jump from point to point. Regarding the few studies that have addressed action-related discriminations, some success has been found in comparing elicited social behavior in response to live versus stuffed models, but that research suggests that motion cues may be irrelevant to animals' behavior (e.g., [18]). Ethological studies of reptiles and birds, however, have found prey motion to be important for predators, with some suggestion that motion and static information are integrated in decision making processes [19], [20]. These studies, however, examined non-biological, non-articulated, translational motion and not articulated animal behavior.

One recent approach using animation software to generate controlled, realistic classes of different behaviors using digital models holds promise for moving beyond these limitations [21], [22]. These studies have suggested that pigeons can learn to categorize different types of articulated locomotion (walking vs. running) depicted in videos by learning a viewpoint-independent temporal sequence of poses using edge-based information involving the entirety of the digital animal model. These results provide some of the best evidence yet that animals can categorize motion or action in a manner resembling humans [14], [15], [23], [24]. Furthermore, given that the digital, locomoting “animals” were different quadruped models, it is unlikely that the stimuli activated innate motor representations in pigeons. Therefore, those studies suggest that actions can be visually categorized without necessarily being embodied in the observer. These behavioral or verb-like categories would allow similar action “tokens” to be grouped together as functional cognitive units, which would aid greatly in classifying familiar and novel motions and behaviors in the same way as proposed for object categories. Such categories can be used to an animal's advantage by not having to learn every agent's specific appearance, gait, or motion idiosyncrasies. Nonetheless, the natural locomotor actions that were previously studied are limited in their usefulness by their relative simplicity, high repetition rate, and short cyclic duration. These stimuli would thus have generated a relatively small number of motion features that could have mediated a generalized representation of the behaviors without fully utilizing behavioral categories.

To address this limitation, the current experiments examined how both pigeons and humans discriminated videos and images depicting two categories of human behavior that were comprised of highly complex sequences of actions. Each species was tested with the same 3D, digital human model performing a series of different “Indian dance” or “martial arts” behaviors. Each species was required to discriminate between the two classes of behaviors, independent of camera perspective, video starting point, and across multiple exemplars. These human behaviors were selected for several reasons. First, we wanted complex behaviors that were extended, irregular, involved multiple limbs, and were not simply repetitive or cyclical. These two human behaviors offered these attributes. Second, we wanted behaviors that could not be performed by the pigeons such that innate or shared motor representations of these behaviors were far more unlikely than the previous locomotion task [21], [22]. Third, we wanted behaviors that momentarily shared similar static poses, so that attention to the dynamic aspects of the stimuli was potentially critical to the discrimination.

Both dynamic “action” cues and static “pose” cues have been proposed to play a role in both human and computer behavior recognition [11]. To examine for the first time how action and pose information contributes to the discrimination and categorization of complex behaviors by pigeons, we presented and tested each behavior in both dynamic and static presentation conditions. The dynamic condition presented an example of each complex behavior as a 20-s video on each trial. The static condition presented a single static frame randomly selected from these videos on each trial. The relative importance of pose and action information to behavior recognition can then be revealed by differences in these conditions. For example, if the dynamic nature of the actions contributes additional information beyond form information, the discrimination of the dynamic condition should be superior to the discrimination of the static condition.

Three experiments investigated the abilities of pigeons and humans to discriminate these complex behaviors. In Experiment 1, four pigeons familiar with a locomotion discrimination [21], [22] were trained to discriminate between a digital human model performing either a martial arts or Indian dance behavior. Several exemplars of each behavior were tested and each was presented from multiple viewpoints (combinations of different camera rotations, elevations and distances). After acquiring the discrimination, their ability to categorize novel exemplars of each behavior was examined. Experiment 2 tested five motion-naïve pigeons in the same conditions to examine the effect of prior experience on the use of motion features in these stimuli. Finally in Experiment 3, groups of human participants were tested with the presentation conditions used with the pigeons, including one group trained with both types of presentations mixed within training, one group trained with only the dynamic condition, and one group trained with only the static condition.

## Methods

### Ethics Statement

Animal and human procedures were approved by Tufts University's Internal Animal Care and Use Committee (protocol #M2011-21) and Institutional Review Board (protocol #1111024), respectively. Human participants gave written consent prior to the experiment.

### Subjects

Nine pigeons (Experiments 1 & 2) and 60 human participants (Experiment 3) were tested. The four pigeons in Experiment 1 were motion-experienced [21], [22]. The five pigeons in Experiment 2 had operant experience, but were naïve with respect to experiencing motion stimuli in operant tasks. The human participants received partial course credit for their participation.

### Apparatus

Experiment 1 tested pigeons in a touchscreen equipped operant chamber that was constantly illuminated by a centrally positioned 28 V houselight (except during timeouts). Stimuli were displayed on a computer monitor (NEC LCD 1525X; 1024×768, 60 Hz refresh rate) recessed 8 cm behind the touchscreen (EZscreen EZ-150-Wave-USB). Mixed grain was delivered as food reinforcement using a food hopper (Coulbourn Instruments) accessible beneath the touchscreen. Experiment 2 used a second operant chamber that was similar to the chamber used in Experiment l, but that chamber had a different monitor (NEC LCD 51V) and touchscreen (EloTouch). The touchscreen in Experiment 1 was much more sensitive, detecting small differences between pecks but also reporting non-pecking, stimulus-directed behavior, but the use of a discrimination index in the analyses simplifies the comparison between these results. Regarding flicker and smooth motion, both of these monitors operate at high frequencies, and in combination with testing under photopic conditions, are likely to have resulted in the pigeons perceiving the stimuli as temporally stable and in motion. The human participants in Experiment 3 were seated in front of a computer monitor (Dell 1907FPt; 1280×1024, 60 Hz refresh) and used a mouse to interact with a two-alternative forced choice task.

### Stimuli

**Figure 1:**
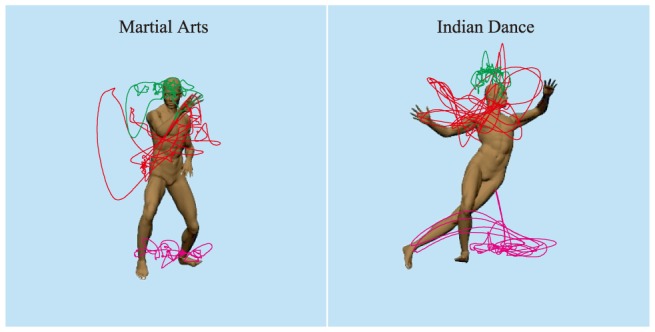
The paths that body parts move through for the two behavioral categories used in this experiment. The green, red, and magenta lines correspond to the trajectories of the head, right hand, and left foot, respectively.

The stimuli were created using a digital human model (motioncapturesociety.com) in Poser 8 (SmithMicro). The motion data for the different exemplars of behavior each were downloaded in the BVH file format from the publicly available database of motion-captured actions at Carnegie Mellon (Indian dance: S. 94 and martial arts: S. 135; mocap.cs.cmu.edu). The martial arts category was generally described as having more defensive, closed, and stable poses than the Indian dance, which more frequently included high-center of gravity and open arms. These motions were clipped for total duration and adjusted so that the figure remained centered in the frame. Four different exemplars, or sequences of poses, of each behavior were used. Each exemplar was rendered from twelve perspectives (combinations of two levels of camera distance, three levels of azimuth, and two levels of elevation). The resulting stimuli continuously varied over each 20-s presentation during each trial at approximately 33 fps (see example motion paths in Figure 1). Each presentation started at a randomly selected frame from the first 300 frames, and after frame 600, the video repeated without delay at frame 1. By starting each trial in the first half of the video, we ensured that this change from frame 600 to frame 1 was not a cue available for discrimination on average until after 13 s of presentation.

### Experiment 1

Four pigeons were trained with two exemplars of each behavior (48 total videos including perspective) for 20 sessions. Pigeons initiated trials with a single peck to a centrally located, white (2.5 cm) ready signal. This signal was then replaced with a discriminative stimulus for 20 s. For S+ stimuli, pecks to the touchscreen during this presentation time were reinforced on a VI-10 schedule, resulting in reinforcement via 2.9 s access to mixed grain anywhere from 0 to 20 seconds after a peck. For S- stimuli, pecks increased a variable dark timeout after the presentations (.5 s per peck). S+ stimuli were also reinforced at the end of the stimulus presentation. For two of the pigeons, the “Indian dance” category was the S+ category, while for the other two, the “martial arts” was the S+ category.

Sessions consisted of 96 trials (48 S+/48 S−) testing equal numbers of the two exemplars of each behavior, with perspective randomly selected for each trial. Forty eight trials tested the *dynamic* condition (24 S+/24 S−), which consisted of a 20 s video presentation of a behavior. Forty eight trials tested the *static* condition (24 S+/24 S−), in which a random frame from one of the videos was presented for 20 s. Twelve randomly selected S+ trials (6 dynamic/6 static) were designated as *probe* trials, in which no reinforcement was delivered. All S+ peck rates were evaluated from these probe trials as they were uncontaminated by food access.

The pigeons were then tested for discrimination transfer to novel exemplars of each behavior. During these six transfer test sessions, eight S− trials and eight S+ probe trials were omitted and replaced by 16 probe trials testing the novel exemplars (96 total trials per sessions). The 16 probe trials (8 dynamic/8 static) evaluated the discrimination with two novel exemplars for each behavior as seen from the closer distance, with both elevations, and at either the 0° and −45° or the 0° and 45° azimuths (tested in alternate sessions).

After transfer testing, all exemplars of both behavior categories were integrated into daily testing. These expanded baseline sessions (48 S−/48 S+ trials – 12 S+ probe trials) tested equal numbers of all four exemplars for each behavior in both dynamic and static conditions. A total of 20 sessions of this type were conducted.

### Experiment 2

Five motion-naïve pigeons were trained to discriminate all four exemplars of each behavior in both dynamic and static presentation conditions. Three pigeons in this experiment were trained with “Indian dance” as the S+ behavior, and two were trained with “martial arts” as the S+. The post-transfer baseline session organization from Experiment 1 was used for acquisition sessions during Experiment 2, except eight S+ trials were designated to be probe trials.

### Experiment 3

Human participants were tested using a two-alternative forced choice task. Each was seated in front of a computer monitor and used a mouse (maximum polling rate of 500 Hz) to indicate their choice on each trial. Minimal verbal instructions were given to the humans in order equate their experience to the pigeons'. Participants were instructed to respond as accurately and quickly as possible. They were informed that correct responses were indicated by a rectangular, centralized green signal presented after each trial and incorrect responses were indicated by a rectangular red signal. No other indication about the purpose of the experiment or the structure of the stimuli was provided. Each trial started when the mouse cursor was moved over the central ready signal. The signal was then replaced by a stimulus display and two laterally displaced white choice squares. Participants indicated their choice by moving the mouse cursor over one of the squares.

Each session consisted of five 64-trial blocks of acquisition training, followed by three 64-trial blocks of testing. During training, all four exemplars of each behavior were used, with equal numbers of each presented in a randomized order. Camera perspective was randomly selected across these trials. The *Mixed* group (n = 20) was trained with both the dynamic and static conditions in a manner directly analogous to the pigeons. The *Dynamic-Only* group (n = 20) was trained with only the dynamic condition, and the *Static-Only* group (n = 20) was trained with only the static condition. The testing portion of the session contained three 64-trial blocks having equal numbers of dynamic and static trials. This was the same for all groups. All participants received both the training and test portions of the session.

## Results

### Experiment 1 – Discrimination acquisition by experienced pigeons

All four pigeons quickly learned to discriminate these digital videos of complex human behaviors. Discrimination behavior was measured using ρ (rho; i.e., as in [25]), which is computed as a normalized Mann-Whitney U. This was calculated from the number of pecks made over the last 10 s of each stimulus presentation during a session, as this time period best measures asymptotic discriminative performance in a trial (e.g., [24], [26], [27]). A ρ of 1 indicates perfect discrimination, with all S+ probe trials receiving more pecks than all of the S- trials within a session, while a chance value of.5 indicates no differential ranking among the conditions.

**Figure 2:**
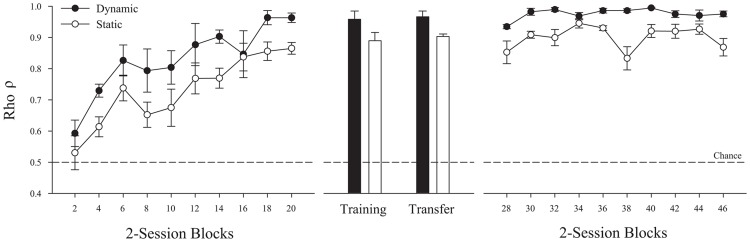
Discrimination performance for the pigeons in Experiment 1 across all three phases of training, testing, and baseline, using ρ (rho) as a discrimination performance metric (see text for more information). Filled symbols represent dynamic performance and open symbols represent static performance. The left panel depicts the successful discrimination acquisition of the behaviors as ρ increased from a chance level of 0.5. The middle panel depicts discrimination during transfer sessions testing two novel exemplars of the behaviors. The rightmost panel shows the pigeons' stable discrimination behavior after the addition of the novel exemplars to daily training. Error bars depict standard error.

The left panel of Figure 2 shows the mean ρ for all pigeons for the dynamic and static presentation conditions over two-session blocks of training. Each bird showed clear evidence of learning the discrimination by the third session. This rapid discrimination was confirmed to be significantly above chance by the second two-session block for both types of presentations (single-mean t-tests – Dynamic *t*(3)  = 11.1, *p* = .002, *d* = 5.6; Static *t*(3)  = 3.6, *p* = .038, *d* = 1.8; an alpha level of.05 was used to evaluate all statistical tests).

Figure 2 also shows that the pigeons exhibited a dynamic superiority effect (DSE) as the dynamic presentation condition consistently supported better discrimination than the static condition over the course of learning. A mixed ANOVA (Presentation Condition × Two-session Block × Behavior Assignment) using ρ was conducted on this training data. It identified a significant main effect of block *F*(9, 18)  = 13.1, *p*<.001, η^2^_p_ = .87, as the pigeons improved over sessions. More importantly, the DSE was confirmed by the significant main effect of presentation condition *F*(1, 2)  = 48.5, *p* = .020, η^2^_p_ = .96. The DSE in this experiment represented an average of 14.9% improvement in ρ with the availability of motion cues when comparing ρ_Static_ with ρ_Dynamic_ (i.e., go/no-go DSE =  [ρ_Dynamic_/ρ_Static_ – 1] ×100%; *SE*_DSE_ = 3.4%). There was no significant effect of whether martial arts or Indian dance was the S+ or S− category (all *F*<1 except presentation mode × behavior assignment *F*(1,2)  = 5.3, *p* = .147). Since the results were consistently the same for both groups, this factor was not included in the later analyses. No other main effects or interactions were significant (all *F*<1). Across all experiments, we also found no significant effects of camera perspective when this variable was analyzed, since the birds discriminated similarly well across perspectives. Consequently, perspective is also not further discussed.

### Experiment 1 – Novel Transfer Testing

The pigeons showed significant transfer of their learned behavior discrimination to videos containing novel exemplars of each behavior. The middle panel of Figure 2 shows mean transfer results for each presentation condition as computed using the S+ and S- peck rates from the training and transfer probe trials. Discrimination transfer in both the Dynamic, *t*(3)  = 25.0, *p*<.001, *d* = 12.5, and Static, *t*(3)  = 46.8, *p*<.001, *d* = 23.4, conditions were significantly better than chance. The DSE observed during training continued to appear as dynamic presentations supported significantly better transfer than static presentations (*M*_DSE_ = 7.1%, *SE*_DSE_ = 2.1%; *t*(3)  = 3.4, *p* = .043, *d* = 1.7). These significant patterns in transfer indicate that these behaviors were discriminated using generalized motion and pose features that could be recognized in new examples of the same behaviors.

### Experiment 1 – Baseline

**Figure 3:**
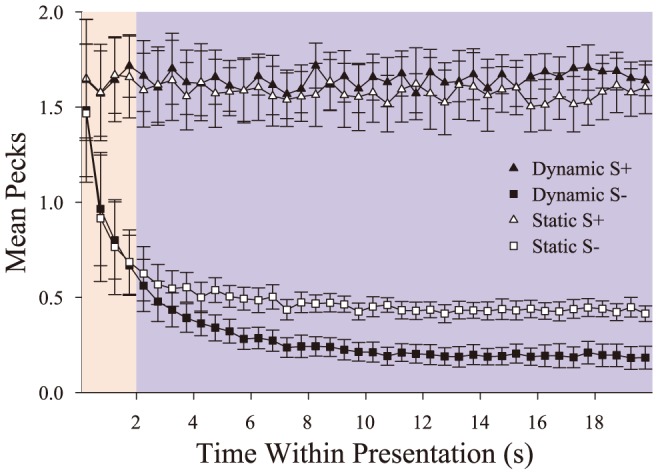
The time course of discrimination within a trial. In this figure, filled symbols correspond to the dynamic trials while open symbols correspond to the static trials. The background colors highlight two hypothesized regions of stimulus processing. The red region highlights that portion of the trial in which primarily static processing supports discrimination, while the blue highlights the portion of the trial where dynamic information assists in discrimination. Error bars depict standard error.

Following transfer testing, we tested the pigeons for 20 additional sessions using all four exemplars of each behavior. As shown in the right panel of Figure 2, this period confirmed the acquisition results, as all pigeons continued to exhibit a significant DSE (*M*_DSE_ = 8.8%, *SE* = 0.79%). Furthermore, we used this period to examine the time course of their discriminative behavior as a function of presentation time within a trial. The results of this analysis are in Figure 3. This figure depicts mean peck rate to the S+ and S− stimuli over the course of a trial (in 500 ms bins) for both dynamic and static presentations. Peck rates started high at the beginning of all presentations. In the case of S+ stimuli, they remained high for the remainder of the presentation. Pecking to the non-reinforced S− decreased across time within a trial. S+ and S− peck rates diverge by 500–1000 ms in a trial (e.g., the red filled region). Testing peck rates in the first second using 250 ms bins, we found this initial difference rapidly and significantly emerged by 250–500 ms, *t*(3)  = 3.7, *p* = .034, *d* = 1.9, after the beginning of a presentation.

The time course of the DSE is also readily seen in these curves, as the separation between S+ and S− peck rates grows larger for the dynamic than the static conditions (see the blue filled region in Figure 3). The dynamic and static S− peck rates significantly diverge at 2 s and this DSE continues to grow until approximately 10 s when peck rates seem to stabilize at different terminal values for these conditions. A repeated-measures (RM) ANOVA (Time [500 ms bins] × Presentation Condition) on peck rate confirmed this interaction was present for S− trials, *F*(39,117)  = 13.7, *p*<.001, η^2^_p_ = .82. An analogous ANOVA on peck rates for S+ trials indicated that a DSE existed on positive trials, as the dynamic S+ peck rate was consistently higher by the end of the trial than the static S+ peck rate, *F*(39,117)  = 1.84, *p* = .007, η^2^_p_ = .38. These differences in peck rates seem to reflect two parts to the pigeons' ongoing discrimination of these videos. S− peck rates during both dynamic and static conditions suppress similarly at the beginning of each presentation, reflecting a quick, early process that likely relies on static cues. At about 2 s, pecking during the dynamic condition further suppresses, suggesting a second, slower phase or process where motion cues begin to exert additional control on behavior, likely delayed by the temporal nature of motion cues to be observed, extracted, and accumulated.

### Experiment 2 – Discrimination acquisition by naïve pigeons

**Figure 4:**
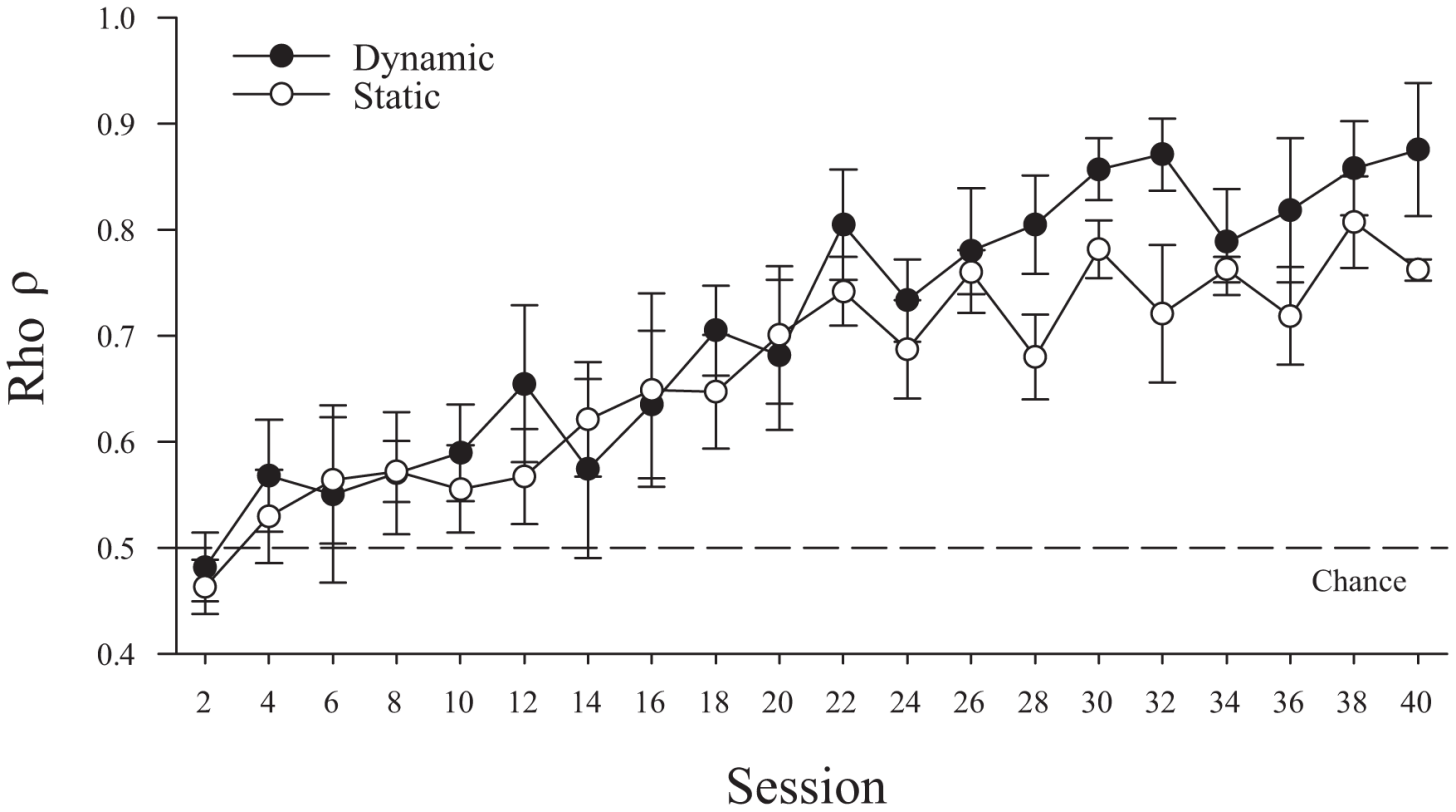
The acquisition of discrimination for five motion-naïve pigeons. Filled symbols correspond to the dynamic trials while open symbols correspond to the static trials. Error bars depict standard error.

Using new pigeons that had not previously been trained with moving stimuli during operant procedures, we tested whether the DSE observed in Experiment 1 was a byproduct of the experimental history of those animals [21], [22]. Figure 4 shows the acquisition of the discrimination by these motion-naïve pigeons in two-session blocks. Initially, the dynamic and static conditions were acquired at similar rates, but after acquiring the discrimination, a DSE was apparent for all five pigeons as the dynamic presentation condition again supported faster learning and better discrimination than did the static presentation condition. A repeated measure ANOVA (Presentation Condition ×2-session Block) on ρ restricted to the first 20 sessions of acquisition (similar to Experiment 1's acquisition phase) revealed a main effect of block *F*(9,36)  = 3.3, *p* = .005, η^2^_p_ = .46, but unlike with the motion-experienced pigeons, there was no main effect of presentation condition or its interaction with session (*F*s<1). Analyses of the remaining twenty sessions of training, did confirm a DSE in these motion-naïve birds with additional experience. Over these latter 20 sessions, an analogous RM ANOVA (Presentation Condition ×2-session block) identified only a main effect of presentation condition *F*(1,4)  = 8.8, *p* = .041, η^2^_p_ = .69. This significant main effect confirms the emergence of a DSE (calculated over last 20 sessions, *M*_DSE_ = 11.7%, *SE*_DSE_ = 3.2%) with the motion-naïve pigeons, too, although it took longer to emerge than with the motion-experienced ones. Thus, the DSE with the motion-experienced birds was not a product of pre-experimental training to attend to motion cues, although this experience may have facilitated how quickly it was exhibited.

To examine the potential benefit of this motion experience, we conducted a mixed ANOVA across experiments (Presentation Condition × Two-session Block × Experiment). For this purpose, the data from Experiment 1's acquisition phase were compared to the first twenty sessions of acquisition for the motion naïve pigeons in the same two-session blocks. The ANOVA indicated that motion experience resulted in better overall discrimination by a main effect of Experiment, *F*(1,7)  = 16.9, *p* = .005, η^2^_p_ = .71. The interaction between Presentation Condition and Experiment approached significance, *F*(1,7)  = 4.9, *p* = .062, η^2^_p_ = .41, as the motion-experienced pigeons seemed to be able to use the motion cues more effectively during this earlier portion of training than the motion-naïve birds.

### Experiment 3 – Human Testing

Most human participants were able to learn the task to criterion within the hour of testing (69%). Nevertheless, to our surprise a number of them did not (31%). To accommodate this difference, we divided the participants into three groups based on performance: learners, late-learners, and non-learners. A *learner* was a participant who achieved above 70% accuracy by the final block of training. A *late-learner* was below 70% accuracy during the final training block, but above 70% by the final block of testing. Finally, a *non-learner* was below 70% accuracy during both the final training block and the final testing block (and often still at chance). These criteria resulted in identifying 12 learners, 1 late-learner, and 7 non-learners in the *Mixed-Training* condition; 13 learners, 2 late-learners, and 5 non-learners in *Dynamic-Only*; and 7 learners, 6 late-learners, and 7 non-learners in the *Static-Only* condition. Despite the trend suggesting the *Static-Only* training was more difficult than the other two conditions, the distributions of learning category was not statistically significantly dependent on the training condition, χ^2^(4)  = 7.0, *p* = .136. Examinations of the protocols conducted after learning suggested that the majority of non-learners were attending to irrelevant, social-like, cues (e.g. head position, body orientation) during their attempts to learn the discrimination. Since all the pigeons learned the discrimination, we focused the remainder of the analyses on the 32 humans classified as learners in order to make comparisons across species.

**Figure 5:**
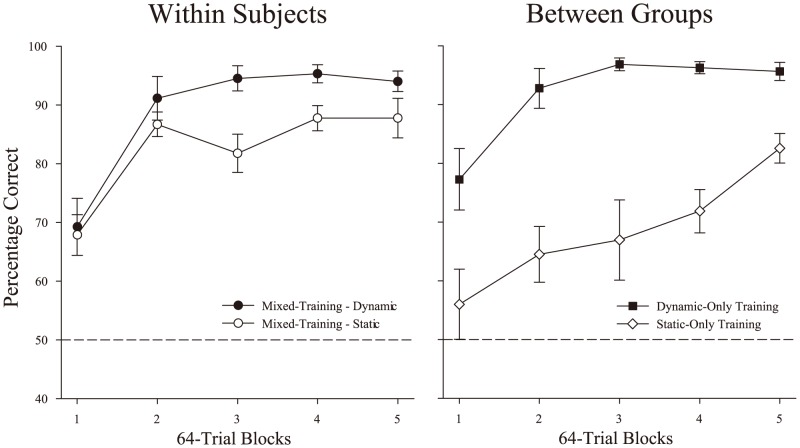
The acquisition of discrimination for 32 naïve human participants across the three groups. The left panel depicts choice accuracy of the *Mixed-Training* condition (circles), separated for dynamic (filled symbols) and static (unfilled symbols) trials. The right panel shows acquisition for the *Dynamic-Only* (squares) training versus *Static-Only* (diamonds) groups. Error bars depict standard error.

The acquisition results for the dynamic and static presentation conditions for the three separate groups of human learners are displayed in the Figure 5. The vast majority were above chance by the second training block (*t*(31)  = 12.68, *p*<.001, *d* = 2.2) indicating that the behavioral categories could be readily discriminated. The left panel of Figure 5 depicts the participants in the *Mixed-Training* task whose experience was most analogous to the pigeons. The right panel of Figure 5 depicts the participants in the two conditions tested only with humans, which permit between-groups comparisons to understand the independent effect of presentation condition on performance. Overall, it appeared the *Dynamic-Only* group learned the discrimination fastest, succeeded by the *Mixed-Training* group, and then followed most slowly by the *Static-Only* group.

The acquisition data for the *Mixed-Training* group was analyzed using a RM ANOVA (Presentation Condition × Five 64-trial Blocks) using choice accuracy. This analysis revealed significant main effects of block, *F*(4,44) = 23.9, *p*<.001, η^2^_p_ = .69, and of presentation condition, *F*(1,11) = 12.3, *p* = .005, η^2^_p_ = .53. There was also a significant presentation × block interaction, *F*(4,44)  = 2.6, *p* = .050, η^2^_p_ = .19. The interaction indicates that the dynamic condition was learned faster and to a higher level of accuracy than the static condition in this group. An average DSE of 8.9% was found between these two conditions over the last block of acquisition.

We next compared performance in the *Dynamic-Only* and *Static-Only* groups. A mixed design ANOVA (*Dynamic-Only* vs. *Static-Only* Group × Block) on choice accuracy revealed significant main effects of block, *F*(4,72) = 18.3, *p*<.001, η^2^_p_ = .50, and group, *F*(1,18) = 32.4, *p*<.001, η^2^_p_ = .64 and a significant group × block interaction., *F*(4,72) = 2.9, *p* = .029, η^2^_p_ = .14. The latter interaction occurred because the *Dynamic-Only* group learned faster and to a higher level of accuracy than the *Static-Only* group. A DSE of 15.8% was found between these two groups over the last block of acquisition, which was about twice the size of that found with the within-subjects comparison.

To better understand the effects of the within and between groups differences, we compared discrimination during the static and dynamic conditions as a function of the type of training. A comparison of the accuracy for the *Static-Only* group and the *Mixed-Training* group's static training trials using a mixed design ANOVA (Group × Block) revealed a significant main effect of training group, *F*(1,17)  = 11.3, *p* = .004, η^2^_p_ = .40, and an interaction between training group and block *F*(4,68)  = 2.6, *p* = .041, η^2^_p_ = .14. An analogous analysis of the dynamic presentations (with the *Mixed* and *Dynamic-Only* groups) revealed no significant differences related to training group, but it did confirm the expected main effect of block, *F*(4,92)  = 32.4, *p*<.001, η^2^_p_ = .59. These results indicate that the *Mixed-Training* group learned to accurately respond to static presentations sooner and ultimately better than their *Static-Only* counterparts, but the mixture of the two presentation conditions during training did not impact the learning of the dynamic condition.

All groups were tested with both dynamic and static displays after the five blocks of acquisition training. First, we focused on the *Dynamic-Only* and *Static-Only* groups' performance with the presentation condition that they had not previously experienced. All groups were significantly above chance at discriminating both dynamic and static displays during the first test block: *t*s(12) >31, *t*s(6) >, 9 *t*s(11) >13 (all *p*<.001). During the first transfer block, the *Dynamic-Only* group's static accuracy was 89.4% (*SE* = 1.3%) and the *Static-Only* group's dynamic accuracy was 95.1% (*SE* = 2.4%). Thus, both the *Dynamic-Only* and *Static-Only* groups were able to transfer their learned discrimination to the untrained presentation type without difficulty. This suggests that both groups had acquired some form of a general *behavior concept* that supported strong transfer regardless of the type of presentation experienced during training. A mixed ANOVA (Group × Presentation Condition × Block) of accuracy over all three test blocks revealed a continued dynamic superiority effect as reflected in a significant main effect of presentation condition, *F*(1,29)  = 37.8, *p*<.001, η^2^_p_ = .57, with a mean DSE of 9.7% (*SE* = 1.7%). There was also a Group × Block interaction, *F*(4,58)  = 5.5, *p* = .001, η^2^_p_ = .27, due to the *Dynamic-Only* group's performance declining slightly late in the testing period.

**Figure 6:**
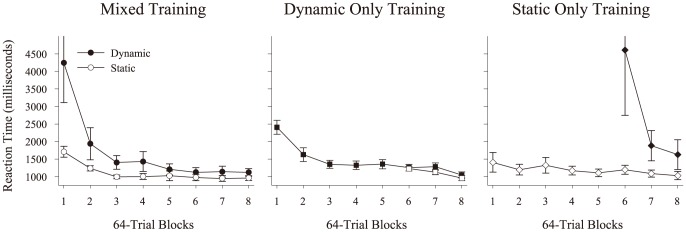
Time to choice response on correct trials for the human participants across training and testing, divided between the three training conditions. Filled symbols indicate dynamic trials, while unfilled symbols indicate static trials. Error bars depict standard error.

Finally, we examined the response time (RT) from stimulus onset to choice. This RT measures a sum of viewing time and choice time. The results for correct responses are presented in Figure 6, divided by training group and presentation condition. The amount of time used to respond during dynamic trials was higher than during static trials, especially during the initial presentations of dynamic displays. As the participants advanced through the blocks of testing, this longer RT on dynamic trials began to decrease and approach, but never became equal to, the RT on static trials. A repeated measures ANOVA (Presentation Condition × Block) using the acquisition data for the *Mixed-Training* group confirmed a significant interaction of presentation condition × block, *F*(4,44)  = 5.7, *p* = .001, η^2^_p_ = .34. This reflects the increased viewing time occurring on dynamic trials relative to static trials and its decrease over time. This interaction was also found when comparing the *Dynamic-Only* and *Static-Only* RTs during the acquisition period using a mixed ANOVA (Group × Block). Here there was the analogous significant group × block interaction, *F*(4,72)  = 6.4, *p*<.001, η^2^_p_ = .26, as the *Dynamic-Only* group initially watched the videos longer than the *Static-Only* group, but these responses became quicker through the experiment. Finally, we analyzed terminal RT over the final block of testing in a mixed ANOVA (Group × Presentation Condition) using only correct responses from all three groups. This analysis revealed a significant main effect of presentation condition *F*(1,29)  = 7.4, *p* = .011, η^2^_p_ = .20, but no effect of group, *F*(2,29)  = 1.6, *p* = .212, or their interaction *F*(2,29)  = 2.5, *p* = .101. Thus, regardless of the original training, dynamic trials were responded to slower than static trials which suggests that additional time was needed or taken to process, perceive, and incorporate motion cues during dynamic trials.

## Discussion

Both the pigeons and the humans learned to discriminate videos depicting categories of complex behaviors based on the non-repetitive, articulated motions of a digital human actor. For both species, the dynamic video presentations of the behaviors promoted superior discrimination in comparison to single presentations of static frames randomly selected from the same videos of the model. Static presentations were also discriminable, however. This *dynamic superiority effect* was present in both species during acquisition and after learning. In the case of humans, this DSE was found through both within-subjects and between-groups designs. The mixture of dynamic and static conditions during training improved accuracy and decreased learning time of the static condition in comparison to training with only static exemplars, but had no detectable effect relative to training with only the dynamic condition. In the case of pigeons, the DSE was found regardless of whether the pigeons had prior experience discriminating simpler locomotive behaviors or not (cf. [26]), although motion experience facilitated learning the complex behaviors, perhaps by increasing attention to dynamic cues. Together, these results suggest that both species used a combination of dynamic action cues and static pose cues to categorize the complex behaviors. This similarity across a mammalian and avian species, given the divergent nature of their underlying visual and motor nervous systems, carries a number of implications for our generalized understanding of behavior recognition.

These results extend the previous action recognition research that tested simpler repetitive behaviors with pigeons [21], [22]. All nine pigeons tested readily learned to discriminate between non-repetitive, human behaviors that were far more complex in their structure and sequencing than the previous walking and running behaviors. This discrimination was also robust and invariant across multiple viewpoints and exemplars and transferred seamlessly to novel examples of each behavior. Further, there was clear evidence of at least two stages in their processing of the dynamic and static presentations.

For the pigeons, the analyses of their peck rates over the course of a trial suggested an early period marked by the rapid emergence of a peck rate difference between the positive and negative instances of each behavior regardless of presentation mode. This early mediation of the discrimination likely reflects the processing of immediately available static pose information during both the static and dynamic conditions. Rapid discrimination of static cues has been seen previously in a shading-based discrimination with pigeons in a similar go/no-go procedure [28]. After a few seconds, a second period emerges where peck rates to the dynamic and static negative conditions further diverge in value. In this case, the dynamic condition shows increased levels of suppression indicative of the processing of additional discriminative information. This subsequent improvement suggests dynamic cues take longer to control the pigeons' discrimination, likely because these motion features require time to develop or integrate into sequential patterns. The time course of these two processes suggests that the discrimination represents a combination of early static cues that are later redundantly enhanced by the addition of dynamic cues, resulting in a DSE.

There is also the possibility that the DSE is not necessarily the direct outcome of motion cues, but instead the result of the pigeon waiting for the “best frame” to make its judgment. This seems unlikely given the time course of the DSE, as it reliably appears within the first few seconds of the trial. However, one possible method to evaluate this alternative is to determine whether any static frames support dynamic-level discrimination. Grouping the 600 frames into 120 five-frame bins, and ignoring for the moment the issue of the different perspectives, results in groups of frames which were seen on average 4.5 times and at most 20 times over the entirety of training and baseline. Examining how each pair of pigeons within the assignment conditions of Experiment 1 responded to negative trials with at least two observations per frame-bin (about 30 of the total 480 bins) suggests unsurprisingly that some frames are better than others for providing information about the behavior of the actors in the video. We were unable to identify, however, single frame-bins that yielded discrimination as good as that observed on dynamic trials. Thus, while a “wait-for-the-best-view” strategy is a possible alternative source for the DSE, given the speed of the DSE and the inability to find static frames as effective as dynamic presentations, it seems unlikely to be the strategy employed by these pigeons. Nevertheless, detailed investigations looking to separate the exact contribution of both specific motions within the videos and specific poses would add to our understanding of the features used by the pigeons for categorization.

Humans, like pigeons, appear to use both pose and action cues in discriminating these stimuli. Humans trained with only dynamic presentations of the behaviors learned significantly faster than those trained exclusively with static presentations, and the humans trained with a mixture of dynamic and static presentations showed this same DSE. Both results indicate the presence and use of additional features in the dynamic condition. Further, over the experiment, humans watched the dynamic videos longer than they did the static frames from those videos, suggesting that an additional information gathering process occurs during dynamic presentations, similar to the pigeons. This “dwell time” could be used to extract motion features from the movement of the actor over time. With experience, less processing time was needed to extract these features as the videos became more familiar. Thus, both pigeons and humans use a combination of dynamic and static cues to discriminate these behaviors, with the static information mediating early fast responding while dynamic information use emerges later to improve the discriminative behavior.

Because groups of humans could be tested with the different combinations of dynamic and static information, it is possible to draw some conclusions about how these conditions may have interacted when presented to the same subject. For humans, the presence of the dynamic information primarily improved the concurrent processing of static displays. One possibility is that seeing the dynamic actions of the model allowed for a better top-down conceptual context for interpreting the more ambiguous pose cues in the static condition. The absence of this top-down categorical information about the different classes of behavior provided by the movements would explain why less than half of the humans trained with only static presentations were able to learn. Relatedly, those humans who learned in the static condition did immediately transfer their learning to dynamic displays (with a notable DSE). Such transfer data indicates that the humans trained with only static information likely inferred a top-down behavioral interpretation of the poses, perhaps from their *a priori* knowledge about the categories of dancing and fighting. Whether a similar representational interaction between the dynamic and static information occurred in the pigeons cannot be ascertained from the current results, but remains an important and compelling aspect of further behavior recognition research.

The contribution of static information to human action processing or recognition is not new. Previous studies have found “action observation” networks are stimulated by static images of implied motion, which rely on observers' understanding of gravity or behavior [29]. A study using a similar technique as the current experiments reported that the areas activated by dynamic videos, but not their static frames, largely corresponded to known, motor-based action recognition networks [30]. What our human results highlight, however, is a relationship between dynamic and static feature processing in categorizing behaviors and that a *behavior concept* can be generated by static cues alone. In contrast to biological motion studies, our full-figured stimuli did not need motion cues to disambiguate the figure's form, allowing the independent contributions of static and dynamic information to be seen. The addition of dynamic cues appears not to enhance species-specific motor networks, but instead helps to extract the configural change over time in articulated motions, regardless of whether the underlying entity is a biological conspecific, a heterospecfic, or an artificial entity [31]. It seems a general property of vision-based action recognition systems whether in humans or pigeons to rely on both static and dynamic features to process form and motion – pose and action – to perceive behaviors.

The fact that both species learned this discrimination and seemed to process the stimuli in similar ways has several implications for our understanding of the potential mechanisms of behavior recognition. A number of motor-based theories of human cognition have been proposed. Implicit in the approach of understanding behavior by the conjoint activation of visual and motor representations (e.g., [5], [32]) is the idea that evolution of species-specific motor-based knowledge is critical to internally simulating or emulating observed actions [7], [9]. The pressures of muscle-powered flight over the last 250 million years of separate evolution, however, has limited the overall size and neural organization of birds in ways that are quite different from mammals [33], [34]. Given that birds and mammals have different organizations of their underlying visual and motor nervous systems, such motor-based theories of behavior recognition are not well supported by the similarities reported in the current comparative results. Pigeons, likely lack any motor representations of human action, especially of complex actions like dancing or fighting. Yet these birds easily discriminated the human actions in these videos, and did so using sequentially similar cues as humans discriminating the same displays. Although humans recognize the behavior of heterospecifics easily, this capacity can be supported by analogy, analytical thinking and language, capacities that not all animals necessarily possess [35]. As a result, theories based on generalized visual mechanisms, as opposed to innate, species-specific embodied action networks, appear better able to account for how these different animals similarly recognize behavior within and across species (for additional critiques of this appoach see [32], [36], [37]). Animals often need to recognize behaviors both within and between classes of animals with which they may share few motor programs, so relying on generalized visual mechanisms specialized for detecting complex articulated motion would provide the most flexible solution.

This vision-based action discrimination of heterospecifics by a bird species provides further insight into the processing ability of collothalamic vision systems. The mammalian visual neuroanatomy results in information lemnothalamically routed from the retina, through the lateral geniculate nucleus, and into the striate cortex, with a secondary processing route that transmits information tectofugally from the retina via the superior colliculus and pulvinar to other telencephalic structures. The avian vision processing system reverses the relative importance of these different pathways, as birds rely on collothalamic processing to perceive the world [34]. Thus, not only can birds perceive and discriminate the actions of non-avian species, but this processing occurs via neural structures different from the mammalian neuroanatomy. Importantly, despite these structural and potentially mechanistic differences, it seems that the use of pose and action cues is critical for this discrimination in both of these phylogenetic classes. This suggests that both pose and action features in the environment are used by different biological visual systems in organizing the visual world [38], [39].

Although pigeons can discriminate these human behaviors, the exact mechanisms supporting this action discrimination have yet to be determined. For example, at least two mechanisms could yield better discrimination with dynamic stimuli over static stimuli. Similar in spirit to the previous “best view hypothesis,” the first mechanism is the activation of multiple pose memories as a result of video playback, resulting in a stronger activation of an action concept. Extracting multiple different “poses” from the dynamic videos or developing many frame-specific associations could result in better overall discrimination during the dynamic trials in comparison to the static trials. This scheme resonates with exemplar memorization theories, as the representation of the action would likely be a collection of snapshots or linked static representations without motion components (usually implicit; cf. [40]). Given that previous research with pigeons has confirmed that coherent change over time results in features that better support recognition of objects and actions [21], [22], [26], this type of representation seems less likely. The second mechanism for the DSE is the redundant availability and use of motion cues during video playback. For this, some memory representation of the optic flow or configural change pattern would need to be stored and compared [27], which agrees with some extant theories of biological motion processing in primates [11] and is being approached by recent ideas of simulation (e.g., [41]).

Finally, these findings are relevant for researchers concerned about the detection of animacy. Detection and processing of animacy in humans, as independent from object processing, has been gaining attention [42], [43]. It is still unclear whether the pigeons exactly perceived an actor engaging in behaviors across all of these conditions. If the pigeons identified the information in these displays as the appearance and motions of acting agents, then this methodology of digital human action sequences provides a paradigm for evaluating the means of agency detection in avian species. The topics of static and motion contributions are also considered in the analysis of animate agents, with some theories arguing for separate regions devoted to processing the relevant static and dynamic information [44]. While studies have explored the neural processing of static and dynamic information in pigeons [45], different stimuli and tasks are generally used for the two domains. Therefore, the methodology of digital acting agents could be valuable for exploring the comparative question of animacy detection and its mechanisms in animals.
